# Hopelessness, individualism, collectivism, and substance use among young rural-to-urban migrants in China

**DOI:** 10.1080/21642850.2014.888656

**Published:** 2014-02-20

**Authors:** Hongfei Du, Xiaoming Li, Danhua Lin, Cheuk Chi Tam

**Affiliations:** ^a^ Pediatric Prevention Research Center, Wayne State University School of Medicine, 4707 St. Antoine, Detroit, MI48201, USA; ^b^ Institute of Developmental Psychology, Beijing Normal University, 19# Xinjiekou Wai Street, Haidian District, Beijing, People's Republic of China

**Keywords:** hopelessness, substance use, individualism, collectivism, migrants, China

## Abstract

The current study aimed to investigate the impact of individualism, collectivism, and hopelessness on substance use. Hopelessness was hypothesized as a mediator between individualism and substance use, and between collectivism and substance use. We tested the hypothesis using a survey of 641 young rural-to-urban migrants in China. Consistent with our hypotheses, individualistic orientation predicted increased hopelessness and subsequently predicted more substance use, whereas collectivistic orientation was associated with decreased hopelessness and subsequently predicted less substance use. Hopelessness fully mediated the relations between individualism and substance use and between collectivism and substance use. The theoretical and practical implications of these findings were discussed.

## Introduction

Substance use including use of alcohol, tobacco, and illegal drugs has been a serious challenge to public health. Numerous studies have documented that substance use is related to sexual behaviour that increases risk for HIV infection (Leigh & Stall, 1993), mental health problems (Elliott, Huizinga, & Menard, 1989), and delinquent behaviour (Flannery, Williams, & Vazsonyi, 1999). Moreover, recent research indicates that substance use is common among vulnerable populations, such as sexual minority people (Hequembourg & Dearing, 2013), street children (Embleton, Mwangi, Vreeman, Ayuku, & Braitstein, 2013), and international migrants (Prado et al., 2009), because these vulnerable populations are more likely to experience social discrimination, have low job accessibility, and encounter barriers to health care. Substance use may be driven by multiple factors, such as cultural backgrounds (Stock et al., 2013), family connectedness and peer affiliation (Prado et al., 2009), and personality traits (Kotov, Gamez, Schmidt, & Watson, 2010).

The issue of substance use has become a major concern in China. The use of alcohol, tobacco products, and illegal drugs has been increasing (Anderson et al., 2006; Chu & Levy, 2005; Cochrane, Chen, Conigrave, & Hao, 2003). One of the vulnerable populations in China is rural-to-urban migrants. The migrant population in China has reported an elevated level of substance use, along with suboptimal health status, depressive symptoms, sexual risk behaviour, and inferior health-seeking behaviour (Chen, Stanton, Li, Fang, & Lin, 2008; Li, Stanton, Chen, et al., 2006; Yang, 2013). Moreover, due to increased modernization and industrialization, rural-to-urban migration has been accelerating in China, which may aggravate the negative impact of substance use. For example, a cross-sectional survey of young sexually active rural-to-urban migrants in Beijing and Nanjing, China, revealed a high prevalence of alcohol use among this population: Approximately one-third of the participants reported to have been intoxicated with alcohol at least once during the past 30 days prior to the survey (Lin et al., 2005). Tobacco use is also a critical challenge to migrants' health. In a study of rural-to-urban migrants in Beijing, China, 51.7% of male participants and 10.9% of female participants reported cigarette use during the past 30 days before the study (Chen et al., 2004). Similar prevalence rates of alcohol use and tobacco use were reported in another study (Chen et al., 2008) with rural-to-urban migrants in China: the 30-day alcohol intoxication rate was 36.7% for males and 16.8% for females; the 30-day cigarette rate was 56.5% for males and 10.5% for females. This study also observed the prevalence rate of lifetime use of illegal drugs: 1.6% for males and 1.8% for females. Therefore, increased knowledge regarding protective and risk factors associated with substance use is urgently needed in order to minimize the prevalence of substance use and develop interventions reducing adverse consequences of substance use in this population.

Personality traits are an influential factor in determining substance use (Sher, Bartholow, & Wood, 2000). Adolescents who had heavier levels of substance use tend to score higher on affiliation, autonomy, exhibition, impulsivity but score lower on achievement, cognitive structure, and harm avoidance (Labouvie & McGee, 1986). Moreover, personality traits related to disinhibition or behavioural undercontrol were associated with substance use disorders (Sher et al., 2000). Researchers suggest that the link between personality traits and substance use can be traced to psychological antecedents of drug use during the earliest years of childhood (Shedler & Block, 1990).

As a dimension of personality, hopelessness refers to negative expectations concerning oneself and his or her future life (Beck, Weissman, Lester, & Trexler, 1974). Adolescents who abandoned hope in uncertain futures were more likely to report substance use, as well as violent and aggressive behaviour and sexual behaviour (Bolland, 2003). The effects of hopelessness on substance use are determined by cognitive elements. Jaffee and D'Zurilla (2009) found that relational problem solving mediated the relationship between hopelessness and substance use: Negative expectations may impair the ability to effectively define problems and, in turn, may result in increased substance use.

The relation between hopelessness and substance use among Chinese rural-to-urban migrants may be influenced by factors that have not yet been identified, given the different cultural, social, and economic environments of migrants compared with their urban and rural counterparts (Li et al., 2009). The present study examined the role of cultural orientation (i.e. individualistic and collectivistic orientations) in affecting hopelessness and substance use among young rural-to-urban migrants in China, which may help explain how cultural orientation is associated with risk behaviour among migrants.

Cultural orientation prescribes social norms and values and includes various dimensions, which can direct and change cognition, attitudes, and behaviour (Hofstede, 1980; Hofstede, 2001; Schwartz, 1994, 2006). A major dimension of cultural orientation is individualism versus collectivism (Du et al., 2013; Oyserman, Coon, & Kemmelmeier, 2002). Individualists tend to pursue self-fulfilment and uniqueness, whereas collectivists emphasize social obligations and group goals. Western cultures (e.g. North America) embrace individualism rather than collectivism, whereas East Asian cultures (e.g. China) value collectivism over individualism. More important, individual differences exist within cultures: a person in a collectivistic culture can be individualistic, and vice versa (Oyserman et al., 2002). Empirical studies have revealed that individual differences in individualism or collectivism within cultures affected psychological well-being (Caldwell-Harris & Ayçiçegi, 2006; Fulmer et al., 2010). For example, for university students in a highly individualistic culture (i.e. Boston), mental-health symptoms were negatively associated with individualism but positively associated with collectivism. In contrast, in a collectivist culture (i.e. Istanbul), mental-health symptoms were positively correlated with individualism but negatively correlated with collectivism (Caldwell-Harris & Ayçiçegi, 2006). Therefore, it is worthwhile to examine the associations of individualism and collectivism with health outcomes.

Regarding the impact of cultural orientation on substance use, existing evidence tends to show that collectivistic orientation is a protective factor, whereas individualistic orientation is a risk factor (Johnson, 2007; Le, Goebert, & Wallen, 2009; Lin, Wu, & Detels, 2011; Sue, Zane, & Ito, 1979). There may be multiple mechanisms through which cultural orientation can influence substance use, such as parental monitoring (Tyson & Hubert, 2002, 2003; Wong, Klingle, & Price, 2004) and peer affiliation (Le et al., 2009).

The current study investigated hopelessness as an individual-level mediator in the relationship between cultural orientation and substance use. Individualism has been found to be positively related to hopelessness because people high in individualism may receive less social support from others relative to those high in collectivism (Scott, Ciarrochi, & Deane, 2004). In addition, depression, a concurrent symptom with hopelessness (Eremsoy, Çelimli, & Gençöz, 2005), was also reported to have a positive association with individualism in both Chinese and American samples (Zhang, Norvilitis, & Ingersoll, 2007). Taken together with the evidence, it seems a reasonable assumption that individualism will increase hopelessness and, in turn, substance use, whereas collectivism will reduce hopelessness and thereby substance use. More important, hopelessness will mediate the relation between cultural orientation and substance use. We surveyed a community sample of young rural-to-urban migrants in China to test these hypotheses.

## Method

### Data source and study sample

The data of the present study were derived from the baseline survey of a larger intervention study aiming at reducing HIV risk among young rural-to-urban migrants (under 30 years of age) in China. The study initially recruited 660 rural-to-urban migrants in China but 19 of them were excluded from the current analysis because they reported an age >30 years, leaving 641 participants to test these hypotheses. The intervention study was conducted in Chaoyang District in Beijing, the capital city of China. Beijing had a permanent resident population of 19.6 million at the time of the study (Beijing Bureau of Statistics, 2010). Chaoyang District, the most populous district in Beijing, had a population of 3.64 million, including about 840,000 migrants at the time of the study. To ensure the representativeness of the sample, we adopted a venue-based recruitment procedure (Li et al., 2004; Li, Stanton, Chen, et al., 2006) and recruited the young migrants from their workplaces (e.g. shop, club, factory, and construction site), migrant settlements, streets, and job markets. All participants were migrants who have moved from rural areas to Beijing at least three months earlier and have not yet been granted permanent Beijing residence. China has strict household registration (“hukou”) policies so that migrants find it hard to obtain permanent urban residence. Most of the migrants returned to their home village after they worked in the city for a period of time (Li, Stanton, Fang, & Lin, 2006). Without permanent residence, the migration population is strongly stigmatized and experience mental-health symptoms (e.g. depression, anxiety, social isolation) (Li, Stanton, Fang, et al., 2006). For this study, we obtained ethical approval from Wayne State University and Beijing Normal University Institutional Review Boards.

### Measures

Individualistic and collectivistic orientations were assessed with the cultural orientation scale (Chirkov, Ryan, Kim, & Kaplan, 2003). Of the total 24 items in the scale, 12 measure individualistic orientation (e.g. “To rely on oneself most of the time and rarely rely on others”) and the other 12 assess collectivistic orientation (e.g. “To maintain harmony within any group that one belongs to”). Participants responded to each item on the 5-point Likert scale ranging from 1 (strongly disagree) to 5 (strongly agree). Individualistic orientation and collectivistic orientation were indicated by mean scores of 12 items of each subscale, respectively. Higher scores indicate higher levels of individualistic or collectivistic orientation. Both subscales demonstrated good reliability (individualistic orientation, *α* = 0.86; collectivistic orientation, *α* = 0.91).

Hopelessness was assessed with a 5-item scale developed by Whitaker, Miller, and Clark (2000). Of the 5 items, 3 items measure expectation about the future (i.e. “Sometimes I feel there is nothing to look forward to in the future”; “I just live for today”; “It's really no use worrying about the future, because what will be will be”) and 2 items measure loss of motivation (i.e. “Sometimes I feel that I'm being pushed around in life”; “I have little control over the things that happen to me”). Participants responded to each item on a 4-point Likert scale ranging from 1 (strongly disagree) to 4 (strongly agree). An average score of the 5 items was used with higher scores indicating higher levels of hopelessness (*α* = 0.81).

Substance use was assessed with an 8-item scale on eight types of substance use including tobacco, alcohol, ecstasy, ketamine, methamphetamine, other types of drug (e.g. heroin), drug injection, and needle sharing during drug injection. Participants reported how often they have used each substance in the past six months (1 = never, 2 = occasional, 3 = at least once every month, 4 = at least once every week, 5 = almost every day). An average score of the eight items was used with higher scores indicating more substance use (*α* = 0.81).

Participants reported demographic information including gender, age, marital status, the number of years of living in Beijing, and monthly income.

### Analysis

We conducted analyses of variance or Pearson product moment correlation coefficient to assess the association of demographic characteristics (i.e. gender, age, income, marital status, and length of migration) with substance use. If sample characteristics were significantly associated with substance use, we included them in the subsequent analysis as covariates. A correlation matrix was created to examine the bivariate correlations among individualistic and collectivistic orientations, hopelessness, and substance use. To investigate the role of individualistic and collectivistic orientations on hopelessness and substance use, a path analysis was performed using Mplus 5.1 (Muthén & Muthén, 2008). The model was specified such that hopelessness mediated the effects of individualistic and collectivistic orientations on substance use. To determine the suitability of a model, we used several fit indices: the comparative fit index (CFI), the root-mean-square error of approximation (RMSEA), and the standardized root-mean-square residual (SRMR). A value of CFI close to or greater than 0.95, a value of RMSEA close to or less than 0.06, and a value of SRMR close to or less than 0.08 reflect adequate fit of a model to the data (Hu & Bentler, 1998). The indirect effect was examined using bootstrapping procedure with 5000 bootstrap resamples (Preacher, Rucker, & Hayes, 2007). An indirect effect was considered significant in case zero was not contained in the 95% confidence intervals.

## Results

Demographic characteristics of the participants are presented in Table 1. Among the 641 participants, 376 are male migrants and 265 are female migrants. The age range of the participants was from 17 to 30 with a mean of 24 years. Overall, they had been working in Beijing for an average of 3.62 years and their average salary was ¥2455.81 (around $378) per month, which was much lower than ¥4201.25 (around $647), the average salary of the whole population in Beijing at the time of the study (Beijing Statistical Information Net, 2010). Approximately 60% of the participants have not been married.

**Table 1.** Demographic characteristic of the sample.

Variables	Overall	Male (376)	Female (265)	*t* and *χ*^2^
Age (SD)	24.11 (3.30)	24.52 (3.32)	23.52 (3.17)	3.84***
Years of being a migrant worker in Beijing (SD)	3.62 (2.51)	3.89 (2.65)	3.22 (2.25)	3.19**
Mean monthly income in Yuan (SD)	2455.81 (1223.69)	2544.99 (1335.56)	2324.44 (1025.82)	2.28*
*Marital status* (%)				
Unmarried	59.60	55.50	65.40	7.98*
Unmarried but living together	7.50	9.30	4.90	
Married	32.60	35.00	29.30	
Divorced, widowed, or separated	0.30	0.30	0.40	

*p < 0.05.

**p < 0.01.

***p < 0.001.

Preliminary analyses showed a significant difference in substance use by gender, (*F*(1, 639) = 21.97, *p* < 0.001), such that males reported more substance use (*M* = 1.42, SD = 0.58) than females (*M* = 1.21, SD = 0.48). Substance use was significantly associated with age, (*r* = 0.08, *p* < 0.05), monthly income, (*r* = 0.13, *p* < 0.01), but not with length of migration, (*r* = 0.02, *p* = 0.71). Substance use did not differ by marital status, (*F*(3, 625) = 1.68, *p* = 0.17).

**Figure 1.**
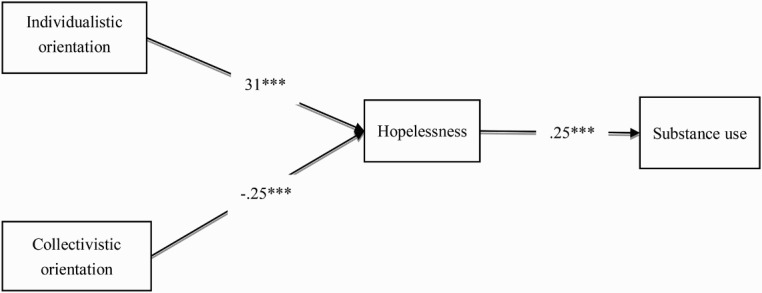
Path model examining the mediational effect of hopelessness. ***p < 0.001. CFI = 0.96; RMSEA = 0.06; SRMR = 0.02.

Table 2 presents the correlations and descriptive data of individualistic orientation, collectivistic orientation, hopelessness, and substance use. The path model (Figure 1) provided a good fit, (*χ* ^2^ = 6.06, df = 2, *p* = 0.05, CFI = 0.96, RMSEA = 0.06, and SRMR = 0.02), explaining 11% of the variance in substance use. As hypothesized, hopelessness significantly predicted substance use, (*β* = 0.25, *p* < 0.001). Individualistic orientation had a significant effect on hopelessness, (*β* = 0.31, *p* < 0.001) and a significant indirect effect on substance use, through hopelessness, (*β* = 0.08, *p* < 0.001), 95% confidence interval, 0.03–0.15. Similarly, collectivistic orientation had a significant effect on hopelessness, (*β* = −0.25, *p* < 0.001), and a significant indirect effect on substance use, through hopelessness, (*β* = −0.07, *p* < 0.001), 95% confidence interval, −0.13 to −0.03. Neither individualistic orientation (*β* = 0.01, *p* = 0.93) nor collectivistic orientation (*β* = −0.02, *p* = 0.63) had a significant direct effect on substance use. Moreover, gender (male = 0; female = 1) (*β* = −0.21, *p* < 0.001) and age (*β* = 0.01, *p* < 0.05) significantly predicted substance use. However, income did not predict substance use and was removed from the model.

**Table 2.** Means, standard deviations, and intercorrelations for measures.

Variables	1	2	3	4
1. Substance use	–			
2. Individualistic orientation	−0.01	–		
3. Collectivistic orientation	−0.04	0.76***	–	
4. Hopelessness	0.23***	0.11**	−0.04	–
*M*	1.33	3.41	3.59	2.19
SD	0.55	0.60	0.65	0.56

**p < 0.01.

***p < 0.001.

## Discussion

As expected, individualism increased hopelessness and greater hopelessness increased substance use, whereas collectivism decreased hopelessness and less hopelessness predicted reduced substance use. A path analysis confirmed our a priori hypothesis that hopelessness would mediate the relation between individualism, collectivism, and substance use. The study findings highlight the importance of cultural orientation in influencing health behaviour and the importance of hopelessness in mediating the association between cultural orientation and substance use.

Hopelessness has been considered as a risk factor for health for a long time. Past research has extensively examined the role of hopelessness in psychological well-being, depression, and suicide intent and behaviour (Abramson, Metalsky, & Alloy, 1989; Du & King, 2013; Kovacs & Garrison, 1985; McMillan, Gilbody, Beresford, & Neilly, 2007). So far, however, not many studies have focused on the impact of hopelessness on other risk behaviour such as substance use (Bolland, 2003). The results in the current study, consistent with previous findings (Bolland, 2003; Jaffee & D'Zurilla, 2009), indicated that greater hopelessness was associated with more alcohol, tobacco, drug use (i.e. ecstasy, ketamine, methamphetamine, and other types of drug), drug injection, and needle sharing during drug injection. In addition, our findings corroborate and complement prior evidence showing the positive relationship between substance use and depression and suicidality (Esposito-Smythers & Spirito, 2004).

Hopelessness is an important mechanism through which cultural and social factors influence substance use. The impact of cultural norms and values on health behaviour can be exerted in many ways, such as parental influence (Lin et al., 2011) and peer affiliation (Le et al., 2009). The current finding indicates that the effects of cultural orientation on substance use can be achieved through personality traits, which corroborates previous findings that personality traits take a significant role in health behaviour (Jaffee & D'Zurilla, 2009). Further research is needed to examine other types of personality related to hopelessness (e.g. loneliness, anxiety) in the relation between cultural orientation and substance use.

Consistent with our hypothesis, collectivistic orientation served as a protective factor, whereas individualism acted as a risk factor for substance use. The protective effect of collectivistic orientation on substance use may be related to cultural backgrounds of the current migrant sample. A person whose cultural orientation matches the prevalent orientation of other people in the society may show better mental and physical health (Caldwell-Harris & Ayçiçegi, 2006). Our findings are in line with this person–culture match assumption by showing that, in the Chinese collectivistic culture, people high in collectivism rather than those high in individualism reported less hopelessness and substance use. In addition, being collectivistic is beneficial for people to relieve stress with collectivistic coping, such as family support, intracultural coping, and fatalism (Yeh, Arora, & Wu, 2006). Hence, people high in collectivism may benefit from collectivistic coping more than others and thereby maintain better mental and physical health. Further research may address two interesting issues. First, it remains unclear whether collectivism (or individualism) can serve as a protective factor for substance use among people in individualistic cultures (e.g. North America). According to the person–culture match theory, individualism may protect people from risky behaviours in individualistic cultures. However, existing evidence seems not to support this idea (Johnson, 2007). Additional empirical studies are needed to test this assumption. Second, international migrants moving from collectivistic societies to individualistic societies (or the opposite) may demonstrate more complex relationships between cultural orientation and risky behaviours (Du & Li, 2013). Future work needs to understand the mechanism behind the association between cultural orientation and health behaviour among international migrants.

While our findings highlight important contributions, there are several limitations that warrant attention. The cross-sectional data in the present study limit causal inference. People may first become dependent on substance and then substance use leads people to perceive their future as more hopeless. Self-reports were used in all data collection, which may impair the reliability of the findings. People may feel reluctant to report their history of drug use because drug use is against the law in China. In addition, participants consisted of a convenience sample of rural-to-urban migrants in Beijing, limiting the generalization of findings from the present study to other migrant populations.

Substance use is a serious challenge to public health among not only internal migrants in China, but also international migrants all over the world. To understand the precedents for substance use is beneficial for the development of prevention and intervention programs. The current findings allowed us to explore meaningful pathways that may address health disparities among migrant populations. Future research needs to better address the roles of cultural and social backgrounds and personality traits in risky behaviour.
